# Admission blood glucose and 10-year mortality among patients with or without pre-existing diabetes mellitus hospitalized with heart failure

**DOI:** 10.1186/s12933-017-0582-y

**Published:** 2017-08-14

**Authors:** Osnat Itzhaki Ben Zadok, Ran Kornowski, Ilan Goldenberg, Robert Klempfner, Yoel Toledano, Yitschak Biton, Enrique Z. Fisman, Alexander Tenenbaum, Gregory Golovchiner, Ehud Kadmon, Alexander Omelchenko, Tuvia Ben Gal, Alon Barsheshet

**Affiliations:** 1 Department of Cardiology, Rabin Medical Center, 39 Jabotinski St., Petah Tikva, Israel; 2 “Leviev” Heart Center, Sheba Medical Center, Tel Hashomer, Ramat Gan, Israel; 3 Division of Maternal–Fetal Medicine, Helen Schneider Hospital for Women, Rabin Medical Center, Petah Tikva, Israel; 4 Sackler Faculty of Medicine, Tel Aviv University, Tel Aviv, Israel

**Keywords:** Heart failure, Diabetes mellitus, Prognosis, Admission blood glucose

## Abstract

**Background:**

High admission blood glucose (ABG) level has been associated with a poor short-term outcome among non-diabetic patients with heart failure (HF). We aimed to investigate the association between ABG levels and long-term (10 years) mortality in patients with or without pre-existing diabetes mellitus (DM) admitted with HF.

**Methods:**

We analyzed data on 1811 patients with DM and 2182 patients without pre-existing DM who were hospitalized with HF during a prospective national survey. The relationship between ABG and 10-year mortality was assessed using the Cox proportional hazard model adjusting for multiple variables. ABG was analyzed both as a categorical (<110, 110–140, 140–200, and >200 mg/dL) and as a continuous variable.

**Results:**

At 10 years of follow-up the cumulative probability of mortality was 85 and 78% among patients with DM and patients with no pre-existing DM (p < 0.001), respectively. Among patients with no pre-existing DM, glucose levels of 110–140, 140–200 and ≥200 mg/dL were associated with 9% (p = 0.140), 16% (p = 0.031) and 53% (p < 0.001) increased mortality risk compared to ABG < 110 mg/dL. Each 18-mg/dL (1-mmol/L) increase in glucose level was associated with a 5% increased risk of mortality (p < 0.001) among patients with no-pre-existing DM. In contrast, among patients with DM, only those with glucose levels >200 mg/dL had an increased mortality risk (>200 mg/dL versus <110 mg/dL; HR = 1.20, p = 0.032).

**Conclusion:**

Among hospitalized HF patients with no pre-existing DM there is a linear relationship between ABG level and long-term mortality, whereas among patients with DM only ABG level >200 mg/dL is associated with increased mortality risk.

## Background

Patients admitted with heart failure (HF) carry a poor long-term prognosis with an estimated 50 percent mortality rate at 5 years of follow-up [1]. Diabetes mellitus (DM) is a major contributor to the development of HF and its associated poor prognosis [2–6]. Several studies have shown that admission blood glucose (ABG) levels are correlated with short-term prognosis in the acute HF setting both in patients with DM and in patients without DM [7–13]; nevertheless, others have shown no correlation between ABG levels and short-term mortality among patients with acute HF [14, 15]. Furthermore, data on the association between ABG levels and long-term mortality among HF patients is scarce [11, 13, 16].

The current study was designed to investigate the association between ABG levels and long-term mortality (10 years) in hospitalized HF patients with DM and without pre-existing DM included in the Heart Failure survey in Israel (HFSIS). Since plasma glucose is an easily measured, inexpensive laboratory test and a potentially modifiable risk factor, ABG levels may have important clinical implications for risk stratification and treatment of patients admitted with HF.

## Methods

### Study population and data collection

Baseline and admission characteristics of patients were extracted from the Heart Failure Survey in Israel (HFSIS) 2003 database as previously described [17]. Briefly, this survey, conducted through March and April 2003, included 4102 patients with a diagnosis of either acute de-novo HF, acute exacerbation of chronic HF, or chronic HF, who were admitted to one of the 25 public hospitals operating in Israel. The criteria used for the diagnosis of HF were symptoms of HF (at rest or during exertion) and objective evidence of cardiac dysfunction at rest [18]. Diagnosis of acute HF or exacerbation of chronic HF was determined by the attending physician based on history, symptoms and physical examination, response to HF therapy, chest radiography, echocardiography, radionuclide studies, cardiac catheterization findings and in-hospital course. Detailed data regarding patient characteristics, in-hospital course and management, pre-hospital and discharge medications and diagnoses were collected and recorded by physicians on pre-specified structured forms. The study was conducted in accordance with the International Conference on Harmonization Guidelines for Good Clinical Practice and the Declaration of Helsinki. The ethics committee at each of the participating hospitals approved the protocol.

We have excluded patients with ABG levels below 70 mg/dL (3.9 mmol/L) or above 600 mg/dL (33 mmol/L) as previously done [7] to ensure a homogenous study population and avoid including patients with possible errors, patients with hypoglycemia or severe hyperosmolar hyperglycemia. Thus, our final analysis included 3993 patients of whom 2182 presented with no pre-existing DM and 1811 presented as patients with DM based on either a history of DM obtained from medical records or the use of anti-diabetic agents on admission. Patients were then sub-divided based on their ABG level similar to previously described thresholds [7, 14, 19]: glucose <110 mg/dL (6.1 mmol/L), 110–140 mg/dL (6.1–7.8 mmol/L), 140–200 md/dL (7.8–11.1 mmol/L) and above 200 mg/dL (11.1 mmol/L).

ABG levels were determined as the first blood glucose test on the day of admission as reported in the medical records. Left ventricular ejection fraction (LVEF) was determined by echocardiography. The LVEF data were collected by medical chart review and recorded only if echocardiography was performed within 1 year prior to or during hospitalization. Out of the 3993 study patients, 2775 had LVEF data. Echocardiography was performed in 1536 patients during index hospitalization. LVEF classes were classified as follows: normal—≥50%; mildly impaired—40 to 49%; moderately impaired—30 to 39%; severely impaired ≤30%. The median (interquartile range [Q1–Q3]) timing of echocardiography was 0 month (0–6 months) prior to index admission.

### Outcome measures

The end point of this study was all-cause mortality. During the 10 years after index hospitalization, mortality was assessed for all patients by matching their identification numbers with the Israeli National Population Registry.

### Statistical analysis

Continuous variables were expressed as median (interquartile range). Baseline characteristics of the groups (continuous data) were compared by use of the non-parametric Kruskal–Wallis test; categorical variables and frequencies were compared by means of the χ^2^ test. Kaplan–Meier survival curves were produced and compared using the log-rank test. To examine the relationship between ABG level and mortality several models were conducted. First, potential variables (identified in previously published studies [20, 21] as risk factors for mortality) were evaluated by univariate analysis and were selected based on clinical and statistical significance. The values used to dichotomize the variables were taken from previous reports [20, 21]. Second, multi-variable Cox proportional hazards regression analysis was used to assess the independent association between ABG levels and the risk of all-cause mortality. Covariates included in the multivariate models were identified using a best subset procedure, choosing among a wide variety of available baseline measures with the additional stipulation that they needed to be statistically significant with an individual p value <0.05 for inclusion. Thus, all models were adjusted for ABG (analyzed as a categorical variable and as a continuous variable in separate models), age, chronic obstructive pulmonary disease, active malignancy, urea, creatinine, NYHA functional class III and IV versus I and II, acute de novo or acute exacerbation of chronic HF, systolic blood pressure <115 or ≥115 mmHg, [21] hemoglobin, and the following pre-hospital medication use: statins, β-blockers and furosemide. Gender was also forced into the model. In the subgroup of patients with DM we have repeated the multivariable analysis including also oral hypoglycemic drugs and insulin as covariates. Finally, the same multivariable models for 10-year mortality were adjusted for all of the above parameters plus LVEF class for 2775 patients with LVEF data. All statistical tests were two-sided and a p value <0.05 was considered statistically significant. Analyses were carried out with SAS software version 9.4 (SAS Institute, Cary, North Carolina).

## Results

### Patient characteristics

We identified 2182 patients with no pre-existing DM and 1811 with DM. Patients were sub-divided based on their ABG levels similar to previously described thresholds (Table 1) [7, 14, 19]. The mean follow-up duration was 4.2 years. Patients with DM were younger and more likely to have a history of hypertension, dyslipidemia, ischemic heart disease, stroke and PVD as compared with patients with no pre-existing DM. Patients with no pre-existing DM were more likely to have a history of atrial fibrillation. As for chronic medication use reported on admission—aspirin, statins, beta-blockers, angiotensin converting enzyme inhibitors (ACEIs) or angiotensin receptor blockers (ARBs), furosemide and thiazides were more frequently used in the DM group. Out of 1811 DM patients, 375 patients were treated with insulin and 1013 were treated with oral hypoglycemic medications. The median ABG level was 114 mg/dL (interquartile range 98–140) and 182 mg/dL (interquartile range 134–255) for the subgroups of patients with no pre-existing DM and those with known DM, respectively (p < 0.001). Hemoglobin and GFR levels were lower in the DM group.

**Table 1:** Patient characteristics in the DM group and the no pre-existing DM Group (categorized by quartiles of ABG levels)

	All study patients		No pre-existing DM patients by ABG categories			
	DM patients (n = 1811)	No pre-existing DM (n = 2182)	Glucose < 110 mg/dL (n = 985)	Glucose 110–140 mg/dL (n = 676)	Glucose 140–200 mg/dL (n = 373)	Glucose > 200 mg/dL (n = 148)
Age (y)	73 (65–79)	77 (68–84)^††^	76 (67–83)	76 (67–83)	76 (67–82)	73 (66–80)**
Sex, female (%)	45.2	41.9^†^	37.4	44.5	45.3	52.0**
Hypertension (%)	76.1	62.5^††^	61.6	61.2	64.9	68.2
Dyslipidemia (%)	42.5	30.0^††^	27.4	32.1	31.6	33.1
Current smoker (%)	9.4	10.6	10.3	10.8	11.8	8.8
History of smoking (%)	18.9	16.6 ^†^	17.3	15.2	17.7	14.9
Hx of IHD (%)	82.5	75.4^††^	76.0	73.1	74.9	82.4
S/P MI (%)	41.4	35.9^††^	35.1	36.0	36.5	39.9
acute MI at presentation (%)	27.4	26.2	21.2	29.4	30.0	34.5**
S/P stroke/TIA (%)	14.5	10.7^††^	10.3	10.7	10.7	13.5
AFib (%)	25.2	34.2^††^	34.1	34.8	32.4	36.5
NYHA class III and IV (%)	43.9	38.0^††^	35.3	39.9	41.0	39.7
PVD (%)	12.9	5.8^††^	6.0	6.7	4.8	3.4
COPD (%)	19.6	19.3	17.4	20.9	20.6	21.6
Malignancy (%)	5.4	6.4	5.5	7.3	8.0	4.1
Past malignancy (%)	4.3	4.7	4.1	5.3	5.6	2.7
Active malignancy (%)	0.8	1.0	1.0	1.2	1.1	0.0
Cirrhosis (%)	2.3	3.3	3.5	3.4	2.1	4.7
LVEF (%)^‡^						
≥50	25.1	28.7	28.6	28.6	33.1	18.0*
40–49	21.8	20.8	21.5	22.3	18.1	16.0
30–39	27.2	24.5	25.6	23.0	23.3	30.0
≤30%	26.0	26.0	24.6	26.1	25.6	36.0
Admission vital signs						
SBP, mmHg	142 (123–164)	137 (119–160)	136 (119–158)	140 (120–160)	140 (122–161)	140 (120–163)**
Heart rate, beats/min	82 (70–98)	80 (70–98)	80 (67–90)	82 (70–98)	82 (70–100)	85 (74–100)**
Laboratory values at admission						
Blood glucose mg/dL	182 (134–255)	114 (98–140)^††^	96 (87–103)	124 (117–132)	163 (151–180)	263 (229–319)**
Urea, mg/dL	50 (34–80)	44 (30–65)^††^	45 (30–67)	46 (31–70)	47 (31–71)	50 (34–78)**
Creatinine, mg/dL	1.2 (0.9–1.8)	1.2 (0.9–1.6)^††^	1.2 (0.9–1.6)	1.2 (0.9–1.6)	1.2 (0.9–1.7)	1.2 (1–1.7)*
eGFR (ml/min/1.73 m^2^)	53 (35–74)	56 (40–75)^††^	57 (39–77)	57 (38–73)	55 (38–75)	52 (37–72)**
Sodium, mg/dL	138 (135–140)	139 (136–141)^††^	139 (137–141)	139 (136–141)	139 (136–141)	137 (134–140)**
Hemoglobin, g/L	12 (11–13)	12.5 (11–14)^††^	12 (11–14)	12 (11–14)	12 (11–14)	12 (11–13)
Long term medication use at admission (%)						
Aspirin	64.6	52.7^††^	53.3	52.7	50.9	52.7
Clopidogrel	4.8	4.0	5.0	3.6	3.0	2.7
Statins	42.1	28.7^††^	28.7	28.4	29.2	28.4
Beta blockers	53.7	45.3^††^	46.0	46.0	42.6	43.9
ACE-Is or ARBs	65.2	51.8^††^	51.8	52.2	50.7	52.7
Furosemide	65.0	57.4^††^	58.5	58.4	52.8	56.7
Thiazide	7.45	5^††^	4.0	5.9	6.4	4.0
Spironolactone	15.1	13.6	14.5	12.9	12.6	13.5

Data are given as median (interquartile range) or percentage unless otherwise indicated

SI conversion factors: To convert glucose to millimoles per liter, multiply by 0.055; to convert urea to millimoles per liter, multiply by 0.357; to convert creatinine to micromoles per liter, multiply by 88.4

*ABG* admission blood glucose, *ACE*-*I* angiotensin-converting enzyme inhibitor, *ARB* angiotensin receptor blocker, *COPD* chronic obstructive pulmonary disease, *DM* diabetes mellitus, *LVEF* left ventricular ejection fraction, *MI* myocardial infarction, *NA* not applicable, *NYHA* New York Heart Association, *PVD* peripheral vascular disease, *SBP* systolic blood pressure, *S/P* status post, *TIA* transient ischemic attack

* p < 0.1 and ** p < 0.01 for trend among quartiles of ABG levels in patients with no pre-existing DM

^†^p < 0.1 and ^††^ p value <0.01 for comparison between patients with DM and patients with no pre-exisiting DM

^‡^Of 3993 patients, 2775 had LVEF data

Of note, in the subgroup of patients with no pre-existing DM, elevated ABG levels were associated with higher proportion of females, increased rates of myocardial infarction at presentation, poorer renal function, lower sodium levels and faster heart rates on admission.

### Diabetes mellitus and long term mortality

**Fig. 1:**
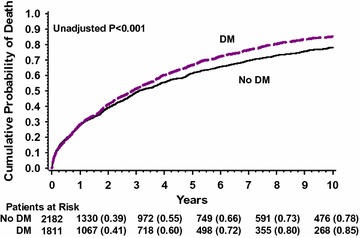
Kaplan–Meier estimates of probability of death in patients with DM versus patients with no pre-existing DM. *DM* diabetes mellitus

The cumulative probabilities of death over 10 years of follow-up in patients with DM versus patients with no pre-existing DM are presented in Fig. 1. By multivariate analysis, patients with DM had a 1.3-fold increased risk of mortality (95% confidence interval 1.22–1.41, p < 0.001).

### ABG and mortality among patients with no pre-existing diabetes mellitus

**Fig. 2:**
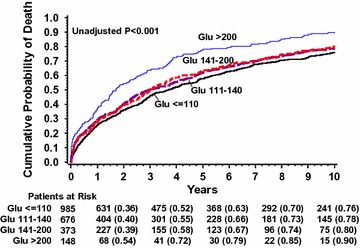
Kaplan–Meier estimates of probability of death in patients with no pre-existing DM according to ABG level subgroups. *ABG* admission blood glucose, *DM* diabetes mellitus

Among patients with no pre-existing DM the 10-year mortality risk correlated with ABG levels (Fig. 2). Thus, at 10 years of follow-up the cumulative probability of death was 76% for patients with ABG <110 mg/dL, 78% for patients with ABG 110–140 mg/dL, 80% for patients with 140–200 mg/dL and 90% for patients with ABG ≥200 mg/dL (p < 0.001 for the overall comparison).

Consistently, multivariate analysis (Table 2) demonstrated that high ABG levels were associated with an increment risk of death. Patients with ABG levels of 110–140, 140–200 and ≥200 mg/dL had a respective 9% (p = 0.140), 16% (p = 0.031), and 53% (p < 0.001) increased mortality risk compared to patients with ABG <110 mg/dL. Also, the results were consistent when further adjusted for LVEF class (among patients who had LVEF data). In a separate multivariable model (Table 2), each 18-mg/dL (1-mmol/L) increase in glucose level was associated with a 5% increased risk of mortality (p < 0.001); notably, ABG measured as a continuous variable remained an independent predictor of mortality also after excluding patients with ABG levels >200 mg/dL (HR = 1.04 per 18 mg/dL increase in ABG, p = 0.014). In a secondary analysis adjusting also for IHD, MI at presentation, antiplatelet agents and thiazides, results remained the same. In addition, results remained the same when patients with acute MI at presentation were excluded from the analysis. There was no significant interaction between ABG and acute or chronic HF status (ABG-by-acute HF p for interaction = 0.837) and there was no significant interaction between ABG and age (ABG-by-age ≥75 p value for interaction = 0.277), implying that the relationship between high ABG levels and mortality does not change according to acute or chronic HF status or in the different age groups.

**Table 2:** Admission blood glucose levels and mortality among patients with no pre-existing DM by multivariate analysis

			HR (95% CI)^†^	p^†^
ABG level category*	Not adjusted to %LVEF	110–140 mg/dL	1.09 (0.97–1.22)	0.140
		140–200 mg/dL	1.16 (1.01–1.33)	0.031
		>200 mg/dL	1.53 (1.26–1.85)	<0.001
	Adjusted to %LVEF^‡^	110–140 mg/dL	1.03 (0.89–1.19)	0.682
		140–200 mg/dL	1.26 (1.06–1.48)	<0.001
		>200 mg/dL	1.50 (1.18–1.90)	<0.001
Per 18 mg/dL glucose (1 mmol/L)*	Not adjusted to %LVEF		1.05 (1.03–1.08)	<0.001
	Adjusted to %LVEF^‡^		1.05 (1.03–1.08)	<0.001
	Patients with ABG level <200 mg/dL only		1.04 (1.01–1.08)	0.014

To convert glucose to millimoles per liter, multiply by 0.055

*ABG* admission blood glucose, *CI* confidence interval, *DM* diabetes mellitus, *HR* hazard ratio, *LVEF* left ventricular ejection fraction

* Adjusted for age, sex, COPD, NYHA 3,4, acute on chronic and chronic HF categories, statins, beta-blockers, furosemide, active malignancy, hemoglobin, urea, creatinine, SBP

^†^HR and p-value compared to ABG < 110 mg/dL

^‡^Of 3993 patients, 2775 had LVEF data

### ABG and mortality among patients with diabetes mellitus

**Fig. 3:**
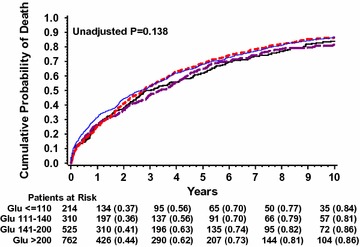
Kaplan–Meier estimates of probability of death in patients with DM according to ABG level subgroups. *ABG* admission blood glucose, *DM* diabetes mellitus

Among patients with known DM, Kaplan–Meier survival curves showed that there were no significant differences in mortality risk between the ABG subgroups (p = 0.138, Fig. 3). Likewise, multivariable analysis (Table 3) demonstrated that there were no significant differences in mortality risk when patients with ABG levels 110–140 mg/dL (HR = 0.91, p = 0.35) or ABG 140–200 mg/dL (HR = 1.08, p = 0.38) were compared to patients with ABG <110 mg/dL. An exception was noted in the ABG >200 mg/dL subgroup experiencing a 1.2-fold increased risk of death (95% CI 1.01–1.42, p = 0.032) compared to patients with ABG <110 mg/dL. When adding oral hypoglycemic medications and insulin to the multivariable analyses, results were consistent showing that among patients with DM only those with ABG >200 mg/dL were associated with increased mortality risk (>200 mg/dL versus <110 mg/dL HR = 1.20, p = 0.04). Adjusted analysis of continuous glucose showed that each 18-mg/dL (1-mmol/L) increase in glucose level was associated with a 2% increased risk of mortality (p < 0.001); this result was mainly driven by the effect of ABG >200 mg/dL as this relationship was statistically insignificant when patients with ABG >200 mg/dL were excluded from the analysis (p = 0.283).

**Table 3:** Admission blood glucose and mortality among DM patients by multivariate analysis

			HR (95% CI)^†^	p^†^
ABG level category*	Not adjusted to %LVEF	110–140 mg/dL	0.91 (0.75–1.10)	0.347
		140–200 mg/dL	1.08 (0.90–1.29)	0.380
		>200 mg/dL	1.20 (1.01–1.42)	0.032
	Adjusted to %LVEF^‡^	110–140 mg/dL	1.05 (0.83–1.33)	0.665
		140–200 mg/dL	1.20 (0.97–1.48)	0.091
		>200 mg/dL	1.36 (1.12–1.66)	0.002
Per 18 mg/dL glucose (1 mmol/L)*	Not adjusted to %LVEF		1.02 (1.01–1.03)	<0.001
	Adjusted to %LVEF^‡^		1.02 (1.01–1.04)	<0.001
	Patients with ABG level < 200 mg/dL only		1.02 (0.98–1.06)	0.283

To convert glucose to millimoles per liter, multiply by 0.055

*ABG* admission blood glucose, *CI* confidence interval, *DM* diabetes mellitus, *HR* hazard ratio, *LVEF* left ventricular ejection fraction

* Adjusted for age, sex, COPD, NYHA 3,4, acute on chronic and chronic HF categories, statins, beta-blockers, furosemide, active malignancy, hemoglobin, urea, creatinine, SBP

^†^HR and p-value compared to ABG < 110 mg/dL

^‡^Of 3993 patients, 2775 had LVEF data

## Discussion

The current study provides two important findings among hospitalized patients with HF. First, ABG level is an independent predictor of long-term (10-year) mortality among patients with no pre-existing DM. Second, among patients with DM only very high ABG levels (>200 mg/dL) predict worse outcome.

The epidemiological association between DM and HF has been firmly established by the Framingham Study [2], which showed a 2.4- and 5-fold increase in the risk of HF in men and women with DM, respectively. Similar findings have been published by others [5, 6, 22]. This may be due to both the presence of co-morbidities associated with DM and to the development of a distinct entity of diabetic cardiomyopathy [23–25]. Functionally, diabetic cardiomyopathy is characterized by increased myocardial fibrosis, higher LV mass and wall thickness leading to both systolic and diastolic dysfunction; the latter is present even before the clinical onset of diabetes, reflecting mainly an increased insulin resistance [26, 27].

Moreover, it has been postulated that HF by itself, and especially in patients with decreased functional capacity, predisposes patients to the development of DM [5, 7, 28, 29].

The strong association between DM and mortality has been demonstrated repeatedly in patients with HF [2–6]. Our results agree with those findings showing a 1.3-fold increased mortality risk in DM patients admitted with HF compared with patients with no pre-existing DM. Furthermore, the association between ABG levels and mortality has also been established in different clinical settings such as in patients with acute coronary syndrome [19, 30–33] and acute cerebrovascular ischemia [34–36]. However, there is a debate whether ABG levels are associated with increased mortality among patients with HF. Studies published so far have mostly focused on the correlation between ABG levels and short-term or mid-term mortality (mostly up to 1 year of follow-up). In their study, Sud et al. [7] included patients who presented to the emergency room with acute HF. The 30-day all-cause mortality was elevated among patients with no pre-existing DM who presented with ABG levels higher than 110 mg/dl. These results are in concordance with previous reports by others [8–10, 12, 13] who have also demonstrated increased 30-day mortality in HF patients with elevated ABG levels. Nevertheless, several investigators have shown opposite results: Kosiborod et al. have investigated the correlation between ABG levels and mortality in a large cohort of elderly Medicare beneficiaries in the USA [14]. Also, in a retrospective population-based study Novack et al. [15] have evaluated the relationship between routine ABG levels of patients admitted for decompensated HF and prognosis. Both studies did not demonstrate a correlation between ABG levels and 30-day or 1-year mortality. In a recently published report from the prospective European Society of Cardiology Heart Failure Long-Term Registry (ESC-HF-LT) [12] an elevated ABG level was not associated with an increased 1-year mortality both in patients with or without DM. Furthermore, in a report from the PROTECT study [35] an elevated ABG level was associated with a slightly better outcome in 180 days of follow-up.

The present study is among the first ones to characterize the relationship between ABG and long-term (10-year) mortality in patients admitted with HF and to show a direct correlation between ABG levels and long-term mortality in patients with no pre-existing DM. We have previously shown [10] that patients with no pre-existing DM admitted with acute HF exhibit an increased in-hospital and 60-day mortality associated with elevated ABG levels; the current study extends these findings to 10 years of follow up. Herein, we included both acute and chronic HF patients and showed that each 18 mg/dL (1 mmol/L) increase in ABG level was associated with an adjusted 5% increased risk of mortality (p < 0.001) among patients with no pre-existing DM. By contrast, among patients with DM, only those with ABG levels ≥200 mg/dL had an increased mortality risk (>200 mg/dL versus <110 mg/dL, HR = 1.20, p = 0.032). Similarly, Newton et al. [13] investigating the relationship between ABG and mortality among 479 hospitalized HF patients during a mean follow-up of 3.4 years, found that ABG greater than 180 mg/dL in patients with no pre-existing DM was a predictor of death, but in patients with DM the level of ABG did not predict mortality.

It is unclear whether high ABG level in HF patients is a marker or a mediator for adverse cardiovascular outcome. As simply a marker for high-risk patients, elevated ABG levels could signify an excess stress response mediated by neuro-hormonal system activation, specifically cortisol and catecholamines [37]. Furthermore, impaired myocardial performance (such as in HF patients) results in the activation of compensatory neuro-hormonal systems, including activation of the sympathetic nervous system [38, 39]. This in turn increases insulin resistance and at the same time stimulates both gluconeogenesis and glycogenolysis [40, 41]. Alternatively, hyperglycemia per-se may mediate the cascade of HF by several independent mechanisms. First, hyperglycemia promotes anaerobic myocardial metabolism, inhibits production of nitric oxide and increases the production of reactive oxygen species in endothelial and vascular smooth muscle cells, thus impairing endothelial function [42, 43]. Second, hyperglycemia alters cardiac structure through post-translational modification of extracellular matrix, both leading to impaired relaxation and increased ventricular stiffness and alters calcium metabolism [44–48]. Finally, insulin deficiency is associated with increased lipolysis and excess circulating free fatty acids, which are toxic to ischemic myocardium and were shown to be associated with the development of arrhythmias [49]. In this context, it should be remembered that HF in diabetic patients has specific biomarkers, different than in their non-diabetic counterparts; only in the former hs-troponin T and the IL1 receptor ST2 are independently associated with both all-cause and cardiovascular outcomes [50, 51].

In contrast to the linear relationship between ABG level and 10-year mortality among patients with no pre-existing DM, we show here that there were no significant differences in the cumulative mortality rates among the ABG subgroups in patients with DM, except for increased mortality risk observed in the multivariate analysis model in patients with very high ABG levels (>200 mg/dL). There are few possible explanations for this unequivocal correlation between ABG level and long-term mortality in patients with DM: As most of DM patients are treated with glucose-lowering medications it is possible that anti-hyperglycemic treatment may obscure the relationship between ABG and clinical outcome at moderate-high ABG levels, but not at very high ABG levels. Alternatively, it is possible that ABG level is a less sensitive marker of activation of the sympathetic nervous system and other neuro-hormonal systems among patients with known DM who already have a significant microvascular or macrovascular disease.

## Study limitations

Study results can be influenced by differences in disease assessment and documentation patterns at participating hospitals. We do not have data on hemoglobin A1C levels or long term DM status and therefore are unable to comment on the differential impact of acutely versus chronically elevated blood glucose levels. As a result, it is conceivable that some patients with DM may have been classified as no pre-existing DM patients. Furthermore, we have no information regarding medication changes and clinical events following hospital discharge. Also, we do not have data on acute stress markers nor on neuro-hormone levels and are not able to establish a casual relationship between elevated ABG levels and mortality. Notwithstanding, the powerful association between elevated glucose levels and mortality is important and implies the need for further study.

## Conclusions

In the present study we have shown that ABG level is a strong independent predictor of long-term 10-year mortality in hospitalized HF patients with no pre-existing DM. Whereas, only very high levels of ABG (>200 mg/dL) were associated with increased long-term mortality among patients with DM. Measurement of an ABG level, an available and inexpensive blood test, in patients admitted with HF may help clinicians detect a subgroup of patients that carry a poorer long-term prognosis. These patients should be monitored more closely and treated more intensively. It is possible that better glucose control may improve prognosis in HF patients as previously suggested in critically ill patients [32, 52, 53]. Further research is needed to determine whether hyperglycemia is a marker or a cause of adverse outcome and whether glycemic control would improve short and long term survival in this high risk HF group of patients.

## Abbreviations

ABG: admission blood glucose
ACEi: angiotensin converting enzyme inhibitor
ARBi: angiotensin receptor blocker inhibitor
CKD: chronic kidney disease
COPD: chronic obstructive pulmonary disease
DM: diabetes mellitus
HF: heart failure
LVEF: left ventricular ejection fraction
MI: myocardial infarction
NYHA: New York Heart Association
